# Near-Real-Time Strong Motion Acquisition at National Scale and Automatic Analysis

**DOI:** 10.3390/s22155699

**Published:** 2022-07-29

**Authors:** Giovanni Costa, Piero Brondi, Laura Cataldi, Stefano Cirilli, Arianna Cuius, Deniz Ertuncay, Piero Falconer, Luisa Filippi, Simone Francesco Fornasari, Veronica Pazzi, Philippe Turpaud

**Affiliations:** 1 SeisRaM Working Group, Department of Mathematics and Geosciences, University of Trieste, Via Eduardo Weiss 4, 34128 Trieste, Italy; costa@units.it (G.C.); pbrondi@units.it (P.B.); laura.cataldi@phd.units.it (L.C.); cirilli@units.it (S.C.); s264024@stud.units.it (A.C.); dertuncay@units.it (D.E.); pfalconer@units.it (P.F.); simonefrancesco.fornasari@studenti.units.it (S.F.F.); pturpaud@units.it (P.T.); 2 Civil Protection Department, Presidenza del Consiglio dei Ministri, Via Vitorchiano 4, 00195 Rome, Italy; luisa.filippi@protezionecivile.it

**Keywords:** Italian Strong Motion Network, seismic hazard, emergency management, Antelope software, strong motion parameters, ground motion parameters, automatic earthquake detection, earthquake monitoring, civil protection, ground shaking maps

## Abstract

A strong motion monitoring network records data that provide an excellent way to study how source, path, and site effects influence the ground motion, specifically in the near-source area. Such data are essential for updating seismic hazard maps and consequently building codes and earthquake-resistant design. This paper aims to present the Italian Strong Motion Network (RAN), describing its current status, employment, and further developments. It has 648 stations and is the result of a fruitful co-operation between the Italian government, regions, and local authorities. In fact, the network can be divided into three sub-networks: the Friuli Venezia Giulia Accelerometric Network, the Irpinia Seismic Network, and all the other stations. The Antelope software automatically collects, processes, and archives data in the data acquisition centre in Rome (Italy). The efficiency of the network on a daily basis is today more than 97%. The automatic and fast procedures that run in Antelope for the real-time strong motion data analysis are continuously improved at the University of Trieste: a large set of strong motion parameters and correspondent Ground Motion Prediction Equations allow ground shaking intensity maps to be provided for moderate to strong earthquakes occurring within the Italian territory. These maps and strong motion parameters are included in automatic reports generated for civil protection purposes.

## 1. Introduction

The seismic shaking of an area strongly depends on the earthquake source characteristics and magnitude, the seismic waves travel paths, and the local geological stratigraphy (e.g., the presence of soft soils overlaying a rocky bedrock) that modify the seismic wave in terms of amplitude, frequency, and duration [1]. These changes are due to the well-known phenomenon called seismic site response/amplification, which is often responsible for damages to structures and infrastructures [2,3].

Italy is characterised by a high percentage of structures built before 1974 (when for the first time specific technical rules were established for seismic design of structures). Moreover, according to a report of the National Institute of Statistics—ISTAT dated 2015, about 1.01% of the residential constructions built between 1946 and 1990 are in a very bad condition (https://www.istat.it/it/files/2015/12/C18.pdf, last access: 16 June 2022). Consequently, damages caused by an earthquake affect mainly old structures built without engineering design and heritage buildings, usually in historical centres [4,5]. Therefore, it is important in terms of risk reduction to characterise the spatio-temporal occurrence and parameters of earthquakes and derive the seismic local response. This information can be used as the fundamental part of the seismic hazard map, either to create a new map or update the existing maps, and, furthermore, it is essential for building design codes [6]. The reliability of the extracted ground motion parameters is crucial in the analysis for civil defense purposes. Combining well-prepared and updated hazard maps, building codes, and post-disaster models can help to increase the effective civil protection preparation against seismic disasters and response during seismic emergencies. They, in fact, help to reduce seismic risk, which is measured in terms of causalities and economic losses in relation to the hazards and the vulnerability of the area (sensu [7,8]).

Monitoring the strong motions of the ground can be carried out by a seismic network that provides an excellent opportunity to obtain information about the source, path, and the ground motion site effects. Gathered information from a major earthquake can provide critical indications for the civil protection authorities. If the recorded data have good quality, this may lead to a more complete view of the consequences of the event. Furthermore, as the distance between the source and the receiver decreases—in other words, stations are in the near-source area—the precision of the measurement increases. Because of their high dynamic range, accelerometers are less prone to saturation. Thus, they record the motions of the ground without losing any information, especially in the near-field zone. Near-field regions tend to be affected by higher damage due to large amplitude ground motions. It is important to have knowledge about the near-fault motions since, first, there are numerous settlements near active fault lines and, second, near-fault motions may exceed the load capacity of the structures given their large amplitudes.

In the last years, the availability of high-quality strong motion records increased worldwide thanks to the installation of a large number of new, last-generation instruments [9,10,11,12,13,14]. At the moment, force-feedback sensors guarantee a high-quality, durable, flat response curve in the long range of frequencies with a high sampling rate [15]. To have high-quality data, the maintenance and calibration of the instruments must be well-established, along with system design, site selection, and the maintenance of data transmission. Regular visits to the stations and a rapid intervention in case of a failure are mandatory to guarantee the reliability and the completeness of the dataset.

The efficient, fast, and reliable data transmission to data centres is guaranteed by modern technologies. The waveforms are available at the data centres in seconds after the events, in the case of continuously recording stations, or in minutes, in the case of triggered stations. Nevertheless, it is necessary to use a complete and reliable procedure of data analysis at the data centres. Some examples of high density networks can be found both for high seismic hazard countries such as California (USA) [9], China [10], India [11], Japan [16], Taiwan [17], and Turkey [12], and moderate seismic hazard countries such as France [13] and Germany [14]. To obtain reliable results in near real-time, information about the recorded data should be updated rapidly. This is one of the major challenges of the integrated networks operationally.

By using a quasi-real time waveform, information related to the seismic event (e.g., seismic moment—$M_{0}$ and moment magnitude—$M_{w}$) and its effect on the ground where stations are positioned (e.g., the peak ground acceleration, velocity, and displacement (PGA, PGV, and PGD, respectively), Housner intensity ($I_{H}$), Arias intensity ($I_{a}$), and spectral acceleration (SA) at different times, commonly at 0.3 s, 1.0 s, and 3.0 s) can be retrieved [18]. Combinations of this information help scientists and engineers to determine the possible damage to structures and infrastructures and the possibility of triggering other natural phenomena (e.g., landslide, soil liquefaction) [2,4,18,19].

In countries such as Italy, where seismicity is considered as moderate-to-high, it is vital to have well-functioning strong motion networks [20,21,22,23,24]. The Italian National Civil Protection Department (DPC) focused on the improvement of the strong motion network—not only the number of the stations but also the quality of the instruments. Currently, the Italian strong motion network, RAN—Rete Accelerometrica Nazionale (in Italian [25]), has more than 600 stations that are distributed all over the country, especially near the seismically active zones. This achievement has succeeded thanks to the co-operation of Italian governmental bodies and regional and local authorities.

In this paper, the current status of the Italian strong motion network, together with its novelty in terms of the management and analysis of the acquired data and information provided, is presented and discussed. In particular, in Section 2.1, the Italian strong motion network is described, while in Section 2.2 and Section 2.3, the near-real time data analysis procedure and that for the automatic report generated for the civil protection authorities, respectively, are illustrated. These two procedures have been developed and are still improved on demand by the researchers of the Seismological Research and Monitoring group (SeisRaM) of the University of Trieste (UniTS). Finally, in Section 3, some recent findings reached by the SeisRaM researchers thanks to the RAN data as well as the performances of the RAN, also compared with other national networks, are shown and discussed.

## 2. Material and Methods

### 2.1. The Integrated Italian Strong Motion Network

**Figure 1:**
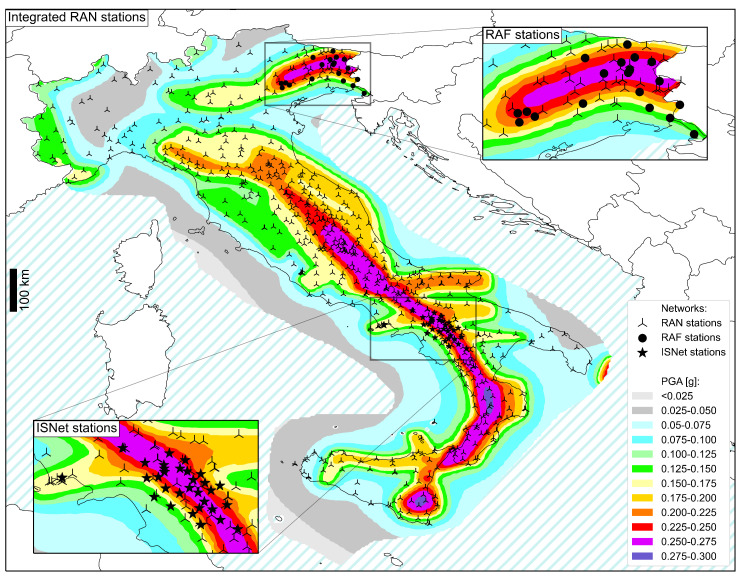
Distribution of the strong motion stations of the RAN (triangle markers) and its tributary networks: the ISNet in the south (star markers, [28] http://isnet.unina.it/, last access: 16 June 2022) and the RAF in the north-east (dots, [27] http://seisram.units.it/, last access: 16 June 2022). The base map is the Italian seismic hazard maps retrieved from http://esse1.mi.ingv.it/d2.html (last access: 16 June 2022), for a return period of 475, i.e., a probability of excess of 10% in 50 years.

The Italian strong motion network evolved to its current state thanks to the inter-institutional agreements among DPC, local and regional authorities, and scientific institutions. The integrated Italian strong motion network is constituted by 3 accelerometric networks: the RAN (triangle markers in Figure 1) owned and managed by the DPC [25,26], the Friuli Venezia Giulia Accelerometric Network (RAF, Rete Accelerometrica Friuli Venezia Giulia, in Italian, [27], dots in Figure 1) in the north-east, owned and managed by UniTS, and the Irpinia Seismic Network (ISNet, [28], star markers in Figure 1) in the south, owned and managed by University of Naples “Federico II”. In the following, the paper focuses only on the RAN and RAF presentation and performance discussion, since the authors are directly involved in the maintenance and management of only these two networks (see Section 2 for details).

**Figure 2:**
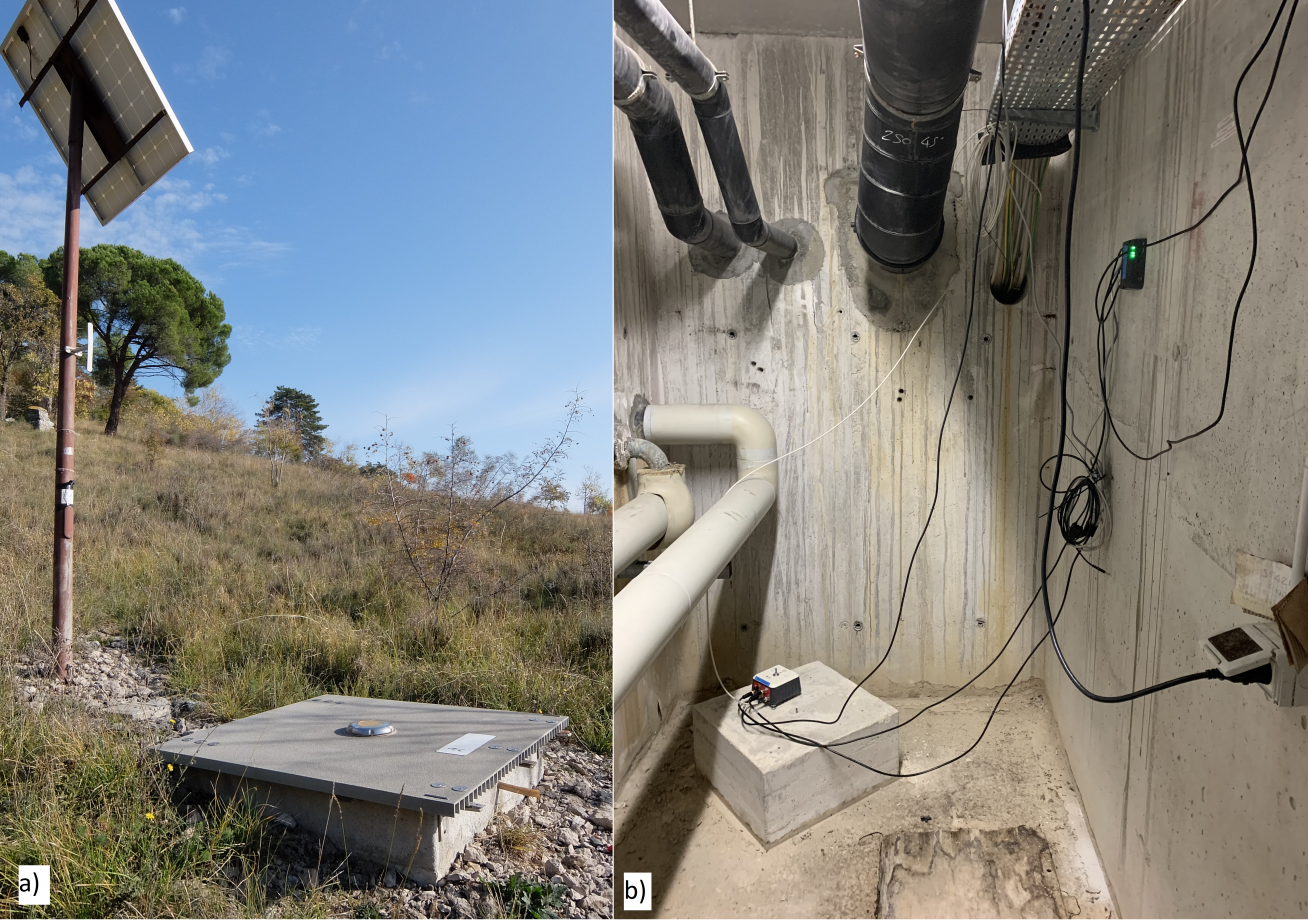
The two typical (**a**) free-field and (**b**) inside-buildings installations used in the RAN network. (**a**) is the the MONF station, while (**b**) is the POR one.

Currently, the network counts 648 points of measures, permanent or temporary, that are more densely distributed within seismogenic areas and mostly in an urban setting. In total, 234 stations are inside sub-stations (mainly little concrete buildings) of an electric power company, while the others are on public land (protected by little fiberglass box) or inside buildings made available by local administrations that provide also the electric power supply needed for the instruments to operate. The permanent stations of the network are fixed, as a standard, on an isolated pillar anchored on rock, if present, or plunged into the sediments, to guarantee the best possible coupling with the ground (Figure 2). The network integrates temporary stations that are installed in the epicentral area on the occasion of an earthquake of a magnitude equal to or larger than 5.0 or of seismic sequences of interest for civil protection. Usually, temporary stations are installed at the basement of public buildings. All the instruments operating on the RAN, made by different Italian and international companies (e.g., Kinemetrics, Reftek, Sara, Solgeo, Syscom, Lunitek), are modern three-component digital accelerometers, and to date 576 stations continuously record and transmit data. As above, the DPC develops annually a plan of ordinary and extraordinary maintenance of the stations in order to ensure a high level (97%) of network efficiency as well as a high level of the single station functionality (each single station cannot be out of order for more than 5 consecutive days).

**Figure 3:**
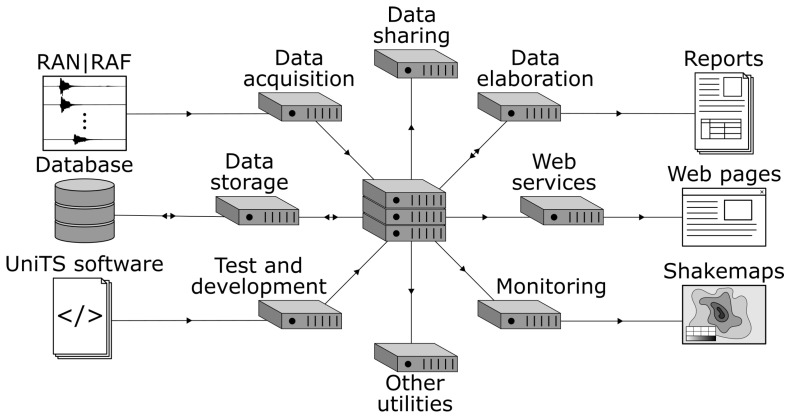
Schema of the RAN Acquisition, Elaboration, Storage, and Data Diffusion Centre (CAED).

At the DPC in Rome (Italy), there is the centre where RAN data are acquired, stored, elaborated, and then diffused. This centre is called CAED, which stands for “Acquisition, Elaboration, Storage, and Data Diffusion Centre” [25,26]. The Antelope real-time system (Boulder Real Time Technology, https://brtt.com/software/, last access: 16 June 2022) has been running at CAED since 2012. It is an integrated suite of programs for data collection and seismic data analysis. It typically runs at the central processing site, where data, metadata, and the results of the data analysis are stored in a relational database and are available for future analyses. In Figure 3 is shown a visualisation of CAED: each operation (acquisition, analysis, parameters calculation, storage, …) runs on a different virtual server. The procedure for the automatic near-real time data analysis is described in detail in Section 2.2.

**Figure 4:**
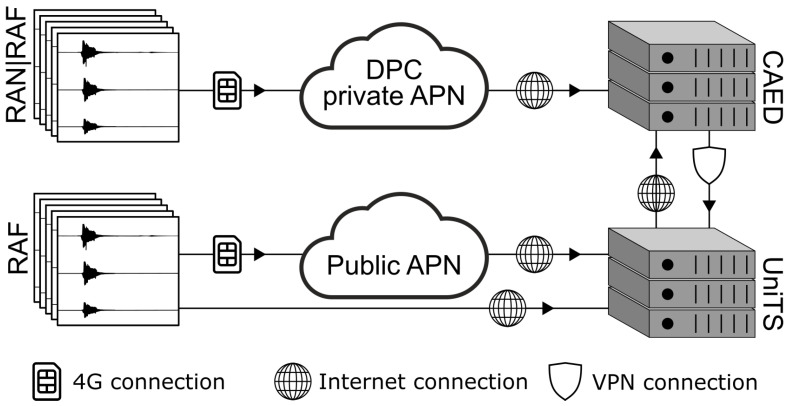
Schema of the Italian ground motion network RAN data transmission from the network to CAED.

An effort has been made by DPC to improve the efficiency of the data transmission from the network to CAED, which is currently part of the DPC IT infrastructure (Figure 4). It uses DPC network connectivity and DPC facilities to communicate over a 3G–4G data transmission system. DPC uses a private access point name (APN), hosted by the mobile phone service provider, for connecting and transferring data from the stations, within a private IP based network. All 3G–4G data traffic is redirected to the DPC network infrastructure, where the RADIUS server, associated to APN, manages the accesses to the private network and associates fix IP addresses to remote devices. The private APN offers a more secure architecture than public APN associated to a SIM card used by remote devices and enables the difficulties related to the management of dynamic public IP addresses to be overcome.

Department of Mathematics and Geosciences of UniTS had the responsibility for the design and evaluation of the packages integrated with the Antelope installation at CAED. To do that, data from stations of the RAF UniTS (IT), Environment Agency of the Slovenian Republic—ARSO (SLO), Austrian Central Institute for Meteorology and Geodynamics—ZAMG (AUS), Croatian Seismological Survey—CSS (CRO), and Italian National Institute for Oceanography and Experimental Geophysics—OGS (IT) are used. These research institutes and the university, together with other institutes from central and eastern Europe, are part of the transnational network called “Central and East European Earthquake Research Network” (CE3RN, http://www.ce3rn.eu, last access: 16 June 2022).

### 2.2. Automatic Near-Real-Time Strong Motion Data Analysis

Rapid seismic data processing and its end products are important for civil protection purposes. They allow authorities in the seismological monitoring area to take action right after the occurrence of a major seismic event, without waiting for manual analysis, which takes at least 15/20 min to provide information about the location and magnitude of the event. Accelerometers can provide reliable information both in near-field and far-field regions that can be used for scientific and civil protection scopes. Having a large quantity of high-quality accelerometric data at the data centre makes the automatic data analysis possible.

When the system operating at CAED finds a new possible location of an event and adds it to the dedicated database, an automatic procedure is triggered. The procedure uses the waveform and the metadata information such as phases and site information from the database and creates a new database to write the results. The results contain information about the seismic source (e.g., $M_{0}$ and stress drop) and about engineering and damage related parameters (e.g., PGA, PGV, and PGD, $I_{H}$ and $I_{A}$, SAs, Integrals of the Squared Acceleration (IA2), of the Squared Velocity (IV2), and of the Squared Displacement (ID2), and the Cumulative Absolute Velocity (CAV)). The waveforms and calculated parameters can be found on RAN’s website (http://ran.protezionecivile.it/IT/index.php, last access: 16 June 2022).

**Figure 5:**
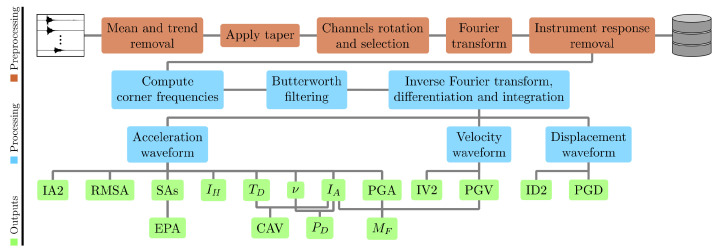
Flowchart of the automatic near-real-time strong motion data analysis implemented at CAED to calculate the source parameters, such as $M_{0}$ and stress drop, and engineering and damage parameters such as PGA, PGV, PGD, $I_{H}$ and $I_{A}$, and SAs.

This procedure, implemented at CAED, is based on the Aspen (Kinemetrics Environmental Monitoring) platform and has been improved and customised by the UniTS researchers. A schematic representation of the procedure workflow is depicted in Figure 5. The main goal of the package is to provide fast, stable, and reliable information on the ground motion parameters from high-quality previously pre-processed data. During the pre-processing of the data, their means and trends are removed as well as possible spikes, a cosine taper (D2 filter) is applied, and finally, the data are corrected for the instrumental response.

The filter procedure automatically selects the frequency range in order to obtain data with a high signal to noise ratio (SNR) over as large as possible of a frequency range. It filters out the frequencies where the SNR is below the threshold [29]. The cut-off frequencies of the filter are searched inside a user-pre-selected frequency range between 0.1 Hz– 50 Hz. A noise window preceding the P-wave arrival and a signal window following the S-wave arrival and whose duration depends on the epicentral distance of the stations is selected, and the spectra are computed using Fast Fourier Transform (FFT). The spectrum is divided into two halves. To choose the minimum frequency of the filter, a smoothed spectrum is selected (three-point running average). Starting from the pre-selected lowest frequency a 50 point sliding window looks for the minimum frequency with SNR>3. The same procedure is applied also for the second half, starting from the highest frequency to the lower ones. The filtering is performed using a zero-phase (applied forward-backward) fourth-order Butterworth filter with corner frequency as found by SNR analysis. Only the waveform data that pass the filtering process are used in the following data processing. This technique allows stable and reliable results to be obtained even for small-magnitude events (M<2). The details about the filtering procedure and the implementation of Andrews’ technique are presented in [29].

Derivation and integration processes are applied to the signals to obtain signals in acceleration, velocity, and displacement domains: the signals obtained are detrended to remove residual noise. PGA, root mean squared acceleration (RMSA), and $I_{A}$ and $I_{H}$ intensities are extracted from the acceleration recordings. The peak spectral accelerations are also computed from the acceleration recordings for 5% damping values at three different periods (0.3 s, 1.0 s, and 3.0 s) and for the complete spectrum. The ratio between the spectral acceleration averaged for the period range 0.1 s– 0.5 s and an amplification factor for the considered damping value is calculated to obtain the effective peak acceleration (EPA). The time lapse corresponding to the 5% and 95% intervals of the total energy is defined as the ground motion total duration ($T_{D}$) [30]. The absolute values of acceleration and the total duration are used to evaluate the cumulative absolute velocity (CAV) [1]. The value of zero-crossing $v_{0}$ is computed and used, along with the $I_{A}$ intensity, to evaluate the destructiveness potential factor (Saragoni index), $P_{D}$[31]. PGV is extracted from the velocity waveform and used, together with PGA and $I_{A}$, to evaluate the Manfredi damage factor, $M_{F}$ [32]. The integrals of squared acceleration (IA2), squared velocity (IV2), and squared displacement (ID2) are evaluated over the total duration of the ground motion (from the corresponding waveforms) [33,34].

The extracted values are also used to automatically produce shaking intensity maps by using ShakeMap [35,36]. The source spectrum is obtained by removing the effects of geometrical spreading and intrinsic attenuation from the FFT of the transverse component of motion, retrieved by rotating the horizontal components of the signal and used to minimise the conversion effects. The velocity and displacement power spectra are obtained from the source spectra, and their integrals are used to compute the $M_{0}$ and corner frequency, $f_{c}$, as in [37]. $M_{w}$ is estimated from $M_{0}$ using the relationship developed by Hanks and Kanamori [38], while the equivalent source radius *r* is computed from $f_{c}$ using Brune’s model [39]. The extracted and computed parameters, both as single station and network average values, as well as the processing information, are stored in dedicated tables.

Events with large magnitude range [27,40] are stored in the UniTS database, which contains strong motion and seismic characteristics. In the database, information about the soil classification from the Eurocode 8 (EC8) [41] and National Earthquake Hazards Reduction Program (NEHRP), along with the geological and geophysical characteristics of the sites (i.e., stratigraphy, morphology, $Vs_{30}$, and $f_{0}$), useful to model the site effect, is also stored.

It is found that the results and processes are consistent [29]. The described package that performs near-real-time strong motion data analysis and that is implemented at CAED has been originally developed by the researchers of the SeisRaM group of UniTS. Currently, this group regularly improves the package by developing new functionalities or by testing the reliability. When a new version is available, before being replaced, the previous version runs in parallel with the new one.

### 2.3. Automatic Near-Real-Time Report to Authorities for Civil Defense Purposes

An automatic reporting procedure has been developed to provide features and information on the potential damage for the observed ground motion. The report is delivered to civil protection authorities and researchers at the University of Trieste via email. The report contains information about the hypocenter, magnitude, and strong motion parameters (see Section 2.2) as well as soil information from the EC8 scale. This information is also sent to the Italian Accelerometric Archive—ITACA (https://itaca.mi.ingv.it/ItacaNet_32/#/home, last access: 16 June 2022). An example of the parameters reported in the automatic reports and related to six different stations (three for each of the two different earthquakes considered) is shown in Table 1. The two earthquakes are the 6 April 2009 L’Aquila (Italy, Lat 42.342, Long 13.38) earthquake ($M_{l}$ 5.6) and the 20 May 2012 Emilia (Italy, Lat 44.896, Long 11.264) earthquake ($M_{l}$ 5.6).

**Figure 6:**
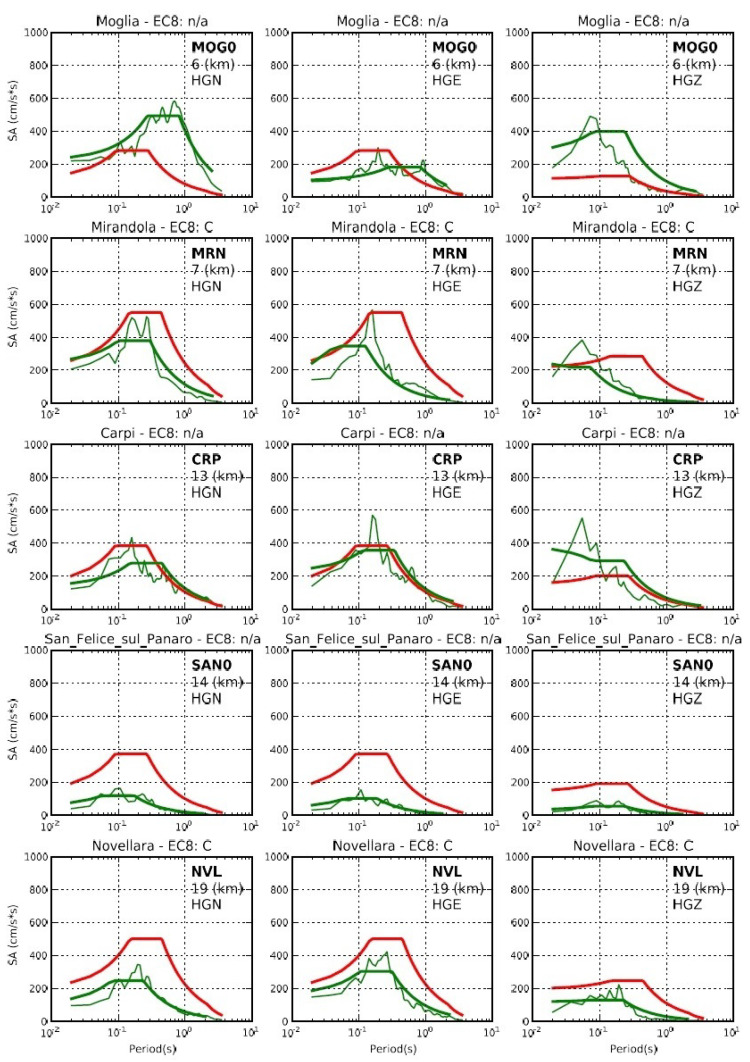
An example of the report page with the comparison of the SA (5% damping ratio given as thin green line), the recorded SA smoothed following the criteria suggested by Microzonation Working Group 2008 guidelines (thick green line), and the predicted SA for a return period of 475 years (i.e., a probability of excess of 10% in 50 years) as in NTC18 (red line) for the 29 May 2012 Emilia earthquake (Lat 44.886, Long 10.967, $M_{l}$5.3, $M_{w}$5.5, Seismic moment $3.92\;\times 10^{17}$Nm, Agency UniTS). In each row are reported the above-mentioned spectra for the three components (HGN, HGE, and HGZ) of the stations located at an epicentral distance between 6 km and 19 km.

Spectral amplitudes (SA) are also provided in the report. SAs are compared with the SA values calculated for the return period of 475 years, i.e., the SA values with a 10% probability of exceedance in 50 years, taken from the Italian Technical norms for buildings (Norme Tecniche per le Costruzioni—NTC18, in Italian, updated by the Ministry for Infrastructure and Transport decree of the 17 January 2018), which is also embedded inside the database. Site corrections are also applied by using the local soil classes, which are mainly from the ITACA database. It is a public database designed to collect, organize, and exchange all the Italian strong motion data recorded by the RAN and all the other local networks available in the national territory and managed by local public institutions or research centres [25]. After the smoothed spectral acceleration is constructed according to the NTC18, it is graphically represented together with the SA. An example relative to the three nearest stations to the epicentre of the 26 October 2016 Castelsantangelo sul Nera (Macerata—Italy) earthquake (Lat 42.898, Long 13.121, $M_{l}$ 6.0, $M_{w}$ 6.2, Seismic moment $5.08\times 10^{18}$ Nm, Agency DPC), the same event and stations as Table 1, is reported in Figure 6.

Once the ground motion parameters have been calculated, for civil protection purposes, it is also necessary to give information about the possible spatial distribution and degree of the expected damage. In the literature, this information is provided by the macroseismic intensity [18,42]. Intensity is a well-known parameter that explains the effects of the ground motion on a given location and structure and on humans. This parameter is easy to explain to general public and widely used by the media to share the effects of a seismic event. The ground motion to intensity algorithm which uses the PGA, PGV, and EPA retrieved from the horizontal components to calculate intensity [43] is implemented in the automatic shakemap creation procedure.

**Figure 7:**
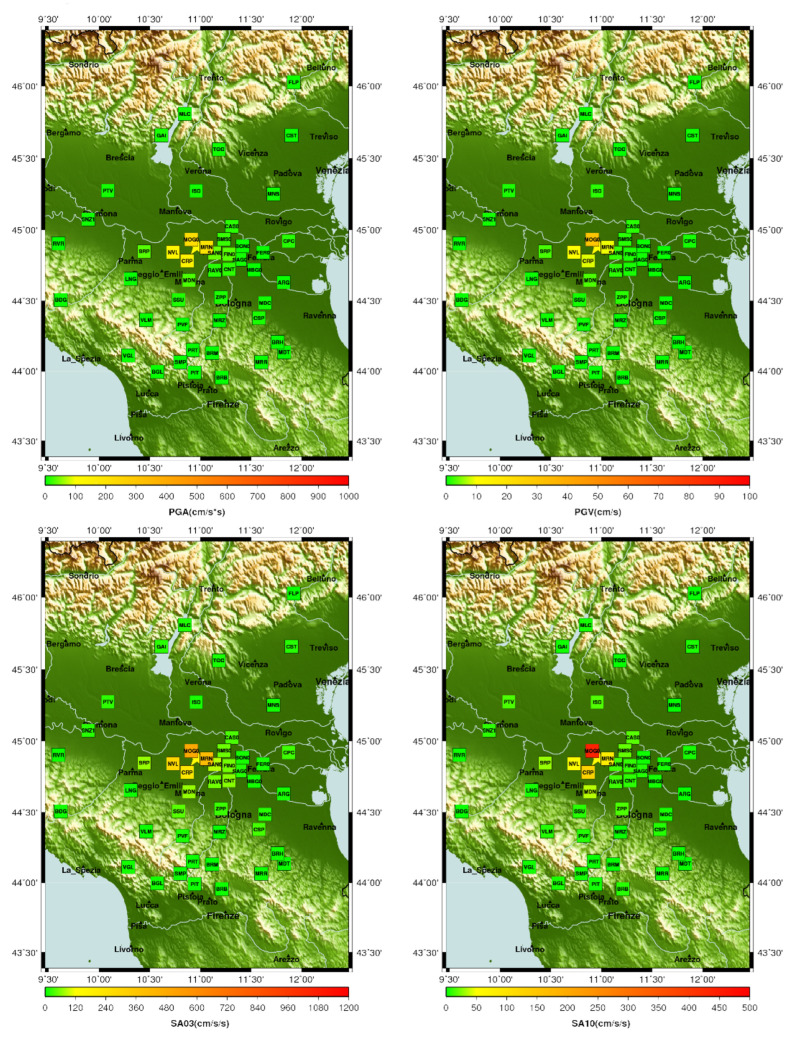
Examples of maps present in the report for the 29 May 2012 Emilia earthquake (Lat 44.886, Long 10.967, $M_{l}$ 5.3, $M_{w}$ 5.5, Seismic moment $3.92\;\times 10^{17}$ Nm, Agency UniTS). In the upper left corner, the peak ground acceleration (PGA); in the upper right corner, the peak ground velocity (PGV); in the bottom left corner, the SA03; and in the bottom right corner, the SA10.

To immediately assess the event, the ground motion parameters values are collected in tables (such as Table 1) and plotted on maps covering the epicentral area. Examples of such maps relative to PGA, PGV, and SAs are shown in Figure 7.

## 3. Results and Discussion

A seismic event’s location and its magnitude are two very important parameters from a seismological point of view, but their assessment takes time (at least 15/20 min for manual inspection). Nevertheless, from a civil protection point of view, having information in real time about the event (and not source) parameters and the shaking of the ground are essential to take fast decisions. This has great importance especially in areas subjected to site effects. Thus, the Italian strong motion network has been designed and implemented to try to fill this gap. It is, in fact, important to remember that the main objective of an accelerometric network is to provide information to calculate the event parameters and not to find event locations. Thanks to the RAN and RAF networks, various scientific studies, with an immediate usability by the civil protection authorities, have been carried out.

One of the main strengths of the RAN resides in the automatic procedure implemented at the CAED that allows researchers both to automatically check and validate the incoming waveforms and to calculate in real time a wide number of ground motion, engineering, and damage related parameters. After an earthquake, to supply a wide range of information to civil protection, the Ground Motion Prediction Equations (GMPEs) are calculated for an extended range of ground motion parameters. From these, the macroseismic intensity estimations are obtained in the epicentral area by using PGA, PGV, $I_{H}$, and information on moment magnitude with PGD. SA03, SA10, and SA30 relationships provide indications to evaluate the response of structures with characteristic periods, respectively, around 0.3 s, 1.0 s, and 3.0 s during an earthquake. IA, IA2, IV2, ID2, and CAV help to model the possible damage of a building, providing indications about the amount of transferred energy and its dissipation per unit of mass during an earthquake. In addition to IV2, GMPE can be exploited to provide further information on moment magnitude, macroseismic intensity, and seismic energy released in the epicentral area [44,45,46]. The ranges of significant recorded values for the above-mentioned parameters are reported in Table 2, while the details of the GMPEs calculation are reported in a paper that is under review in an international journal.

Ertuncay and Costa [47] used the RAN database along with other databases around the globe to develop an algorithm to detect impulsive motions in seismic records. Impulsive motions may occur due to directivity [48] and fling step [49] effects along with local soil conditions [50]. Cataldi et al. [18] proposed a parallel implementation of the instrumental intensity definition, more compliant with the Mercalli–Cancani–Sieberg (MCS) scale. In fact, even if it is common practice to use half-integer intensity values both in macroseismic reports and for instrumental intensity definitions, the MCS scale only defines integer intensity classes. To address this issue, the Naïve Bayes classification methodology could be preferred to the classical linear regression, since it provides a direct probabilistic estimate of intensity as a discrete variable. In the future, this new definition could also be integrated inside the generation of intensity shakemaps [35]. Capabilities of the algorithm were later proven by using data from Turkey [51].

RAN data recorded in north-east Italy are also used to determine the characteristics of the seismic events [52]. To do that, data collected by RAN, along with RAF, OGS, ARSO, Croatian Seismological Network, and ZAMG, are used. A machine learning algorithm is developed by using waveforms along with their FFTs. This study allows researchers to separate quarry blasts from earthquakes and thus to have a better seismic catalog of the region.

RAN data have been recently used to test the performance of a machine-learning approach called ShakeRec and developed by the UniTS researchers to reconstruct ground shaking maps in real-time. The model and its performances are presented in [53]. This model, composed by an ensemble of convolutional neural networks, provides an estimate of specific ground motion parameters and their uncertainty based on the ground motion parameters recorded at the seismic stations, in the form of Voronoi tessellation and a map of the active stations locations, and as a proxy for the site effects. Relying only on data available in real time, ShakeRec can provide useful information for seismic monitoring, filling the information gap between the arrival of the data at CAED and the generation of shakemaps, which relies also on the earthquake magnitude and location.

The current station density of RAN makes the single station functionality still critical at the occurrence of significant earthquake for the Italian territory, especially if the station is in the near field. Nevertheless, the performance of the RAN network, the usefulness of the automatic report, and the automatic procedure to calculate the parameters have been tested in the last years during some strong and intermediate events in Italy. For example, during the 2012 Emilia and the 2016 Amatrice earthquakes, the high number of stations available in the area together with the temporary DPC network installed after the main shock allowed the researchers to assess a high variability in the ground motion near-source characteristics [54,55]. Moreover, as reported in [55] for the Amatrice earthquake, the $M_{w}$ and $M_{l}$ values obtained by the automatic procedure described in Section 2.2 have been compared to those obtained by other research institutes and available online. The result shows that there is a difference of about 0.3 and 0.5 for $M_{w}$ and $M_{l}$, respectively, which is acceptable. The results have direct implications in the development of seismic design codes, in the definition of a seismic hazard, and in seismic spectral design.

**Figure 8:**
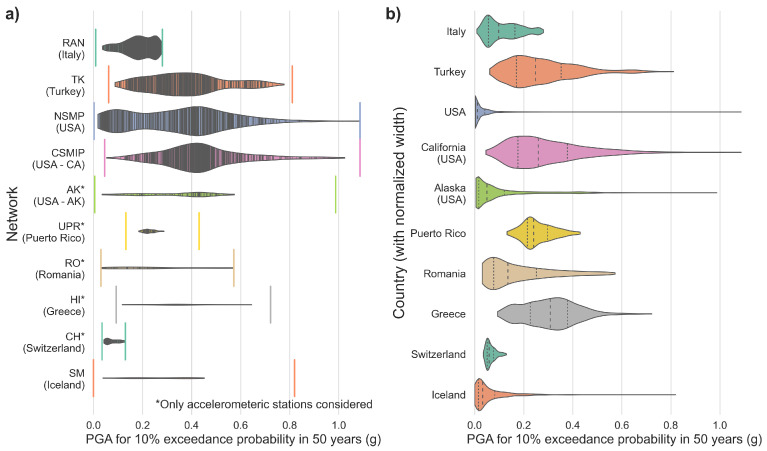
(**a**) Distribution of the accelerometric stations from different strong-motion networks with respect to the seismic hazard values (PGA for 10% exceedance probability in 50 years); (**b**) width-normalised distribution of the seismic hazard values (PGA for 10% exceedance probability in 50 years) in different countries.

To compare the spatial distribution of the accelerometric stations of RAN with other network stations, we use information from the Turkish national strong motion network (TK) [56], the USGS national strong motion program (NSMP), California strong motion instrumentation program (CSMIP), Alaska regional network (AK) [57], Puerto Rico seismic network [58], Romanian seismic network (RO) [59], institute of engineering seismology and earthquake engineering (ITSAK) strong motion network of Greece (HI) [60], national seismic networks of Switzerland (CH) [61], and Icelandic strong motion network (SM) [62] and seismic hazard information from the Turkish seismic hazard map [63], continental USA (used for USA and California) [64], Alaska [65], Puerto Rico [66], and European Facilities for Earthquake Hazard and Risk (EFEHR) for Romania, Greece, Switzerland, and Iceland [67]. The distribution of RAN stations over the Italian territory correlates with its seismic hazard. As seen in Figure 8a, the stations are installed predominantly in the areas with higher seismic hazard for the Italian territory (here, considered as the PGA for 10% exceeding probability in 50 years), in agreement with the goal of the network, which is to monitor strong motions. This specific feature allows us to compare the RAN station distribution with other strong motion networks. Figure 8a shows that RAN is the only network, among those considered, with a station distribution that overlaps with the high values of seismic hazards. Figure 8b shows the (width-normalised) distribution of seismic hazard values for different countries. Even though Italy has a lower maximum PGA with respect to other countries, they are generally more represented in the distribution with respect to other countries. The distributions of the USA and California, with the latter obtained bounding the USA seismic hazard map, are indicative of how the presence of high seismic hazard can be limited to specific regions (with consequences on the distribution of the stations).

To further improve the performances of the RAN, in the future, the number of stations will be increased starting from those areas characterised by a medium level of hazard and a low density of instruments (e.g., the Liguria region in the NW of Italy).

Considering the huge amount of sensors employed in the RAN, a future task could be the development of new versions of the automatic procedure that will allow the following: (a) To calculate more parameters (e.g., the radiated energy will be calculated), (b) To improve the signal to noise ratio (SNR), (c) To perform the analysis of the network anomaly detection (i.e., the identification of events which rise some kind of suspicions) carried out by means of machine learning techniques such as those presented in [68], (d) To improve both directivity and source properties estimation of small to moderate earthquakes [69,70], and (e) To remove, thanks to artificial intelligence, vehicle noise without losing information, implementing hard coded thresholds, or excluding stations [71,72,73,74]. Accelerometric stations, in fact, are usually installed in areas with high population density, and the occurrence of false detection could be quite high.

## 4. Conclusions

This work presents the current situation of the Italian strong motion network called RAN, both from a technical and constructive point of view, as well as the used processing methods. RAN has been improved in terms of emergency response by using the resources of the Italian DPC. Regional seismic networks of RAF and ISNet have been integrated to RAN to improve the number of stations and quality of the seismic network. Currently, the RAN network covers the whole of Italy with densely installed seismic stations with high-quality seismic recorders. The management and maintenance of the networks are carried out by the DPC, and its efficiency allows the DPC to have high-quality data. The data acquisition and the signal pre-processing are provided by the commercial software Antelope, which allows the user to customize their routines according to necessity. Therefore, the Antelope suite that runs at the data-centre in Rome is continuously improved by the UniTS researchers. The procedure implemented by UniTS for the creation of an automatic near-real-time report provides valuable information for the decision making authorities. Indeed, immediately after a strong earthquake, the following information is available: the event origin, the local and moment magnitude, a large set of ground motion parameters, the macroseismic intensity, and relative maps. Both the efficiency of the network, which is above 95% on a daily basis, and the automatic procedure have been tested during the last strong events in Italy.

## Figures and Tables

**Table 1:** Examples of the ground motion parameter tables contained in the near real-time reports for two different events. In the upper part of the table, the parameters for the 6 April 2009 L’Aquila (Italy, Lat 42.342, Long 13.38) earthquake ($M_{l}$5.6) are reported. In the lower part of the table, the parameters for the 20 May 2012 Emilia (Italy, Lat 44.896, Long 11.264) earthquake ($M_{l}$6.0) are reported. Columns are as follows: sta: station; chan: channel; dist: epicentral distance; PGA, PGV, PGD: peak ground acceleration, velocity, and displacement; SA03, SA10, SA30: spectral acceleration (0.3s, 1.0s, and 3.0s); $I_{A}$: Arias intensity; $I_{H}$: Housner intensity; IA2, IV2, ID2: integrals of the squared acceleration, velocity, and displacement; CAV: Cumulative Absolute Velocity, respectively. The three stations considered for the L’Aquila earthquake are: AQA—L’Aquila (EC8 site classification: B), MTR—Montereale (EC8 site classification: B), and SBC—Subiaco (EC8 site classification: A), while for the Emilia earthquake they are: MRN—Mirandola (EC8 site classification: C), MDN—Modena (EC8 site classification: C), and FOR—Forlì (EC8 site classification: C).

sta	chan	dist [km]	PGA [cm/s${}^{2}$]	PGV [cm/s]	PGD [cm]	SA03 [cm/s${}^{2}$]	SA10 [cm/s${}^{2}$]	SA30 [cm/s${}^{2}$]	$I_{A}$ [cm/s]	$I_{H}$ [cm]	IA2 [cm${}^{2}$/s${}^{3}$]	IV2 [cm${}^{2}$/s]	ID2 [cm${}^{2}$s]	CAV [cm/s]
AQA	HGE	9.69	394.41	32.01	5.55	589.84	337.03	44.15	143.68	88.93	$9.96\times 10^{4}$	384.00	28.00	897.70
AQA	HGN	9.69	434.32	26.62	3.62	899.96	234.97	31.62	155.72	76.52	$1.08\times 10^{5}$	429.42	15.81	915.28
AQA	HGZ	9.69	469.36	9.33	1.77	309.31	80.62	17.99	65.30	32.07	$4.51\times 10^{4}$	77.83	5.05	550.35
MTR	HGE	24.48	42.86	3.54	0.79	87.44	58.39	9.44	3.00	14.66	$2.08\times 10^{3}$	24.81	2.53	176.35
MTR	HGN	24.48	61.52	2.89	0.64	157.35	48.68	5.92	5.20	14.83	$3.61\times 10^{3}$	17.27	1.05	216.55
MTR	HGZ	24.48	22.65	3.25	0.92	53.66	30.20	14.40	1.20	11.70	$8.27\times 10^{2}$	14.71	1.91	115.63
SBC	HGE	53.31	2.91	0.48	0.24	6.87	4.88	1.06	0.02	1.42	15.97	0.49	0.21	18.35
SBC	HGN	53.31	3.30	0.65	0.25	8.90	7.07	2.23	0.04	1.80	26.84	0.71	0.23	23.44
SBC	HGZ	53.31	2.50	0.54	0.29	7.84	3.07	1.67	0.02	1.25	14.65	0.59	0.30	17.76
MRN	HGE	18.61	255.30	29.90	7.62	832.37	275.25	49.21	63.60	101.22	$4.41\times 10^{4}$	470.41	47.60	583.36
MRN	HGN	18.61	258.31	46.24	10.35	725.36	549.64	75.75	77.48	157.48	$5.38\times 10^{4}$	1028.72	75.17	624.59
MRN	HGZ	18.61	283.61	5.60	1.52	193.33	42.43	13.35	38.07	20.26	$2.64\times 10^{4}$	32.60	2.82	430.46
MDN	HGE	41.55	36.17	6.42	1.88	69.32	62.70	15.38	3.20	19.19	$2.22\times 10^{3}$	64.51	21.91	249.26
MDN	HGN	41.55	32.75	3.76	1.85	72.44	54.13	9.50	2.44	15.10	$1.69\times 10^{3}$	46.40	17.42	209.91
MDN	HGZ	41.55	28.69	1.51	0.85	76.73	27.99	4.46	1.18	6.52	$8.19\times 10^{2}$	15.91	7.59	129.97
FOR	HGE	99.33	10.40	2.86	2.02	2.02	19.74	16.70	13.20	0.53	10.03	$3.66\times 10^{2}$	49.23	119.57
FOR	HGN	99.33	15.10	2.09	2.10	1.44	22.80	15.40	11.41	0.57	9.03	$3.94\times 10^{2}$	34.54	124.92
FOR	HGZ	99.33	4.81	1.19	1.20	0.98	12.88	8.49	8.09	0.13	4.14	$8.77\times 10^{1}$	12.52	64.08

**Table 2:** Ranges of significant RAN recorded values.

Parameter [unit]	Order of Maximum Value	Parameter [unit]	Order of Maximum Value
PGA [cm/s${}^{2}$]	10${}^{3}$	$I_{H}$ [cm]	10${}^{2}$
PGV [cm/s]	10${}^{2}$	$I_{A}$ [cm/s]	10${}^{3}$
PGD [cm]	10${}^{1}$	IA2 [cm${}^{2}$/s${}^{3}$]	10${}^{6}$
SA03 [cm/s${}^{2}$]	10${}^{3}$	IV2 [cm${}^{2}$/s]	10${}^{4}$
SA10 [cm/s${}^{2}$]	10${}^{3}$	ID2 [cm${}^{2}$s]	10${}^{4}$
SA30 [cm/s${}^{2}$]	10${}^{2}$	CAV [cm/s]	10${}^{3}$

## Data Availability

Data have not been specifically produced for the study. Data sources of Figure 1 and Figure 8 are cited in the paper and can be accessed via data providers.

## Abbreviations

The following abbreviations are used in this manuscript:

APN	Access point name
ARSO	Environment Agency of the Slovenian Republic
CAED	Acquisition, Elaboration, Storage, and Data Diffusion Centre
CAV	Cumulative absolute velocity
CE3RN	Central and Eastern European Earthquake Research network
CSS	Croatian Seismological Survey
DPC	Department of civil Protection
EC8	Eurocode 8
EPA	Effective Peak Acceleration
FFT	Fast Fourier Transform
$f_{c}$	Corner Frequency
GMPE	Ground Motion Prediction Equation
GPRS	General Packet Radio Service
GSM	Global System for Mobile communication
$I_{A}$	Arias intensity
IA2	Integrals of the Squared Acceleration
ID2	Integrals of the Squared Displacement
$I_{H}$	Housner intensity
ISNet	Irpinia Seismic Network
ITACA	Italian Accelerometric Archive
IV2	Integrals of the Squared Velocity
$M_{0}$	Seismic moment
$M_{F}$	Manfredi damage factor
$M_{l}$	Local magnitude
MSC	Mercalli–Cancani–Sieberg scale
$M_{w}$	Moment magnitude
NEHRP	National Earthquake Hazards Reduction Program
NTC	Norme Tecniche per le Costruzioni
OGS	Italian National Institute for Oceanography and Experimental Geophysics
$P_{D}$	Saragoni Index
PGA	Peak ground acceleration
PGD	Peak ground displacement
SA	Spectral acceleration
PGV	Peak ground velocity
RADIUS	Remote Authentication Dial-In User Service
RAF	Friuli Venezia Giulia Accelerometric Network
RAN	Italian Strong Motion Network
RMSA	Root mean squired acceleration
SA	spectral amplitude
SeisRaM	Seismological Research and Monitoring Group
SNR	Signal to noise ratio
$T_{D}$	Total duration
UniTS	University of Trieste
ZAMG	Austrian Central Institute for Meteorology and Geodynamics
